# Conformal carbon nitride thin film inter-active interphase heterojunction with sustainable carbon enhancing sodium storage performance†

**DOI:** 10.1039/d2ta07391a

**Published:** 2022-12-21

**Authors:** Enis Oğuzhan Eren, Evgeny Senokos, Zihan Song, Elif Begüm Yılmaz, Irina Shekova, Bolortuya Badamdorj, Iver Lauermann, Nadezda V. Tarakina, Majd Al-Naji, Markus Antonietti, Paolo Giusto

**Affiliations:** a Department of Colloid Chemistry, Max Planck Institute of Colloids and Interfaces Potsdam 14476 Germany paolo.giusto@mpikg.mpg.de; b PVcomB, Helmholtz-Zentrum Berlin für Materialien und Energie Berlin 12489 Germany; c Technische Universität Berlin Berlin 10623 Germany

## Abstract

Sustainable, high-performance carbonaceous anode materials are highly required to bring sodium-ion batteries to a more competitive level. Here, we exploit our expertise to control the deposition of a nm-sized conformal coating of carbon nitride with tunable thickness to improve the electrochemical performance of anode material derived from sodium lignosulfonate. In this way, we significantly enhanced the electrochemical performances of the electrode, such as the first cycle efficiency, rate-capability, and specific capacity. In particular, with a 10 nm homogeneous carbon nitride coating, the specific capacity is extended by more than 30% with respect to the bare carbon material with an extended plateau capacity, which we attribute to a heterojunction effect at the materials' interface. Eventually, the design of (inter)active electrochemical interfaces will be a key step to improve the performance of carbonaceous anodes with a negligible increase in the material weight.

## Abstract 2

Electrochemical sodium storage of a sustainable carbon anode is further improved *via* a carbon nitride artificial interphase. The heterojunction effect at the materials interface significantly improves the sodium storage performance.

## Graphical abstract

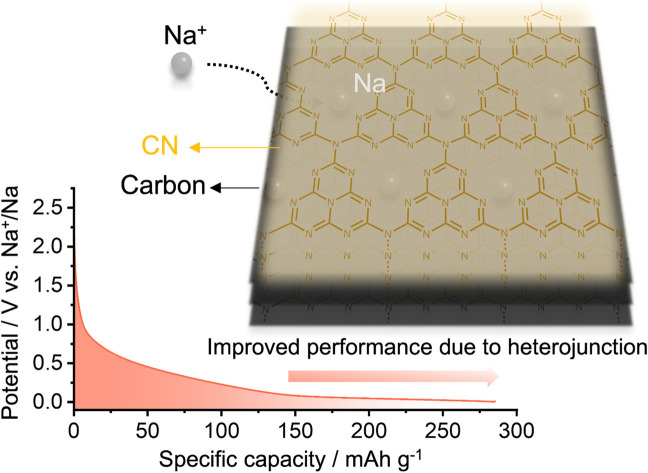

## 1. Introduction

The transition to a more sustainable future of energy storage requires the implementation of modern and affordable electrochemical energy storage systems as soon as possible.^1,2^ Lithium-ion batteries (LIBs) are acknowledged as the current gold standard of secondary battery systems for portable and onboard applications due to their superior electrochemical performance.^3,4^ However, extraction, abundance, and geopolitical concerns regarding lithium and adjunct cathode materials indicate that LIBs are expected to have difficulties meeting the market demand in the medium term.^5^ For these reasons, the search for alternative rechargeable batteries continues unceasingly.

Sodium-ion batteries (SIBs) are considered an up-and-coming alternative as affordable electrochemical energy storage systems for their similar operation principle to the LIBs combined with lower procurement expenses.^6–9^ Still, SIBs are not yet mature enough to replace LIBs commercially due to the elusiveness of the underlying components. Unlike LIBs, graphite cannot be used as an anode material in SIBs as the formation of a thermodynamically stable intercalation compound (*e.g.*, LiC_6_ in LIBs) is not supported.^10,11^ Current studies in SIBs mainly focus on using non-graphitized “hard” carbons (HCs) as the primary anode material. These HCs possess a larger interplanar spacing between the distorted planes compared to graphite and (ultra-)microporosity to enhance the diffusion-controlled sodium storage mechanism, which is a key feature for the existence of a substantial plateau capacity to achieve a high energy density in full batteries.^12–14^

The careful chemical and structural design of HCs is of paramount importance to improve the initial coulombic efficiency (ICE), cycling stability, and rate capability of this class of materials. However, these carbon materials are usually synthesized at very high temperatures (usually between 1200 and 1400 °C),^15^ increasing their energy footprint. Therefore, selecting appropriate precursors and post-modifications are fundamental steps to control the carbons' chemical and structural properties while reducing the processing challenges.^16,17^ An innovative approach for improving the sodium storage performance of disordered carbons relies on post-synthetic processing to deposit an artificial interphase on the active material's surface or a doping agent to tune the electronic structure.^18–23^ Here, the material requirements are different from the ones of electrode (storage) materials. Indeed, such interphases require high chemical and mechanical stability, structural perfection even when applied in low thicknesses, high ionic and low electrical conductivities, and compatible wettability for the electrolyte. In these regards, carbon nitride (CN) is a promising material for artificial interphase due to its low electrical conductivity, high thermal stability, and ionic conductivity, as previously reported.^24–31^ Moreover, CN itself is not an active material for sodium storage,^32^ making it an even better candidate to be employed as a passivation layer. The in-plane structural pores (0.68 nm) might enable the diffusion of sodium ions while simultaneously blocking the negative counter ions.^33^ Our group has recently reported a chemical vapor deposition (CVD) method to coat nm-sized CN thin films homogeneously with tunable thicknesses on target substrates regardless of their shape.^25,31^

Here, we synthesized a carbonaceous anode material from a sustainable source, *e.g.*, sodium lignosulfonate, a byproduct of the paper and pulp industry, denoted as LSC,^34,35^ using a relatively low condensation temperature when compared to widely reported recipes for HCs. Lignin-derived carbon materials have already been exploited as promising electrochemically active materials for various applications such as supercapacitors and batteries.^36–38^ Here, however, we focus on lignosulfonate due to its high sulfur content to generate a “sulfur-doped” carbon, with many advantages discussed in the following. The LSC electrode material was then coated with CN thin films by CVD, where the film thickness was controlled by the amount of precursor, from 10 nm up to 69 nm. This process leads to a significant improvement in electrochemical performance. Exemplarily, the first cycle coulombic efficiency is doubled when compared to the pristine electrode material, and the total specific capacity of the LSC is increased by 30%, with a significant increase in the diffusion-controlled plateau region at lower current densities. Furthermore, the samples coated with the CN show a significant improvement in rate capability. We believe this innovative approach unequivocally sheds light on the importance of designing and controlling the interfaces in carbonaceous anode materials for SIBs.

## 2. Results and discussion

**Fig. 1:**
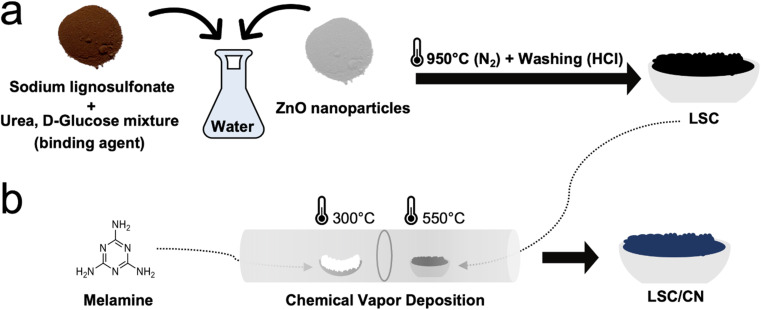
Schematic preparation of LSC and LSC/CN anode materials. (a) Preparation of disordered carbon (LSC) from sodium lignosulfonate precursor and ZnO porogen. (b) Deposition of CN on LSC using the CVD method.

LSC is synthesized using a mixture of lignosulfonate as a carbon source and ZnO nanoparticles as porogen (Fig. 1a). The stepwise thermal treatment allows for condensation and subsequent ZnO carbothermal reduction. In this way, the zinc is eventually removed by evaporation at temperatures above 907 °C (ref. 39) (see ESI† for experimental procedures), and the remaining is washed off with HCl after the thermal condensation. Indeed, inductively coupled plasma optical emission spectrometry (ICP-OES) reveals minor residual of zinc and traces of sodium (Supplementary note 1†). Scanning electron microscopy (SEM) (Fig. S2a†) depicts a porous morphology of LSC, with a typical weakly ordered carbon structure, as confirmed by X-ray diffraction (XRD) and Raman spectroscopy. The XRD pattern (Fig. S2b†) reveals two broad peaks around 23° and 43°, which are attributed to the (002) and (100) reflections of weakly ordered carbons.^40,41^ Exploiting Bragg's law, interplanar spacing of the pseudo-graphitic stacking was calculated as 0.39 nm, larger than that of the natural graphite (0.34 nm), which can be partially attributed to the structural inclusion of sulfur (Table S1 and Fig. S11b†), being too large for the ordinary graphite stacking.^42,43^ From the Raman spectroscopy (Fig. S2c†), G (graphite-like, 1593 cm^−1^) and D (diamond-like, 1349 cm^−1^) bands are used to quantify the order of the carbonaceous materials representing the sp^*2*^ hybridization and the oscillations of the breathing mode, respectively.^44^*I*_D_/*I*_G_ ratio was found as 1.0 from the fitted peak intensity.^44^ The LSC carbon was then introduced into the CVD system and coated with a CN artificial interphase with increasing film thicknesses, as shown in Fig. 1b. As a solid precursor for the CN, melamine was chosen because it is an affordable, non-toxicprecursor and it conveniently sublimates at relatively low temperatures.^45^ Furthermore, melamine is widely used for the synthesis of carbon nitride materials with stoichiometry close to the ideal C/N ratio of 0.75.^31^

**Fig. 2:**
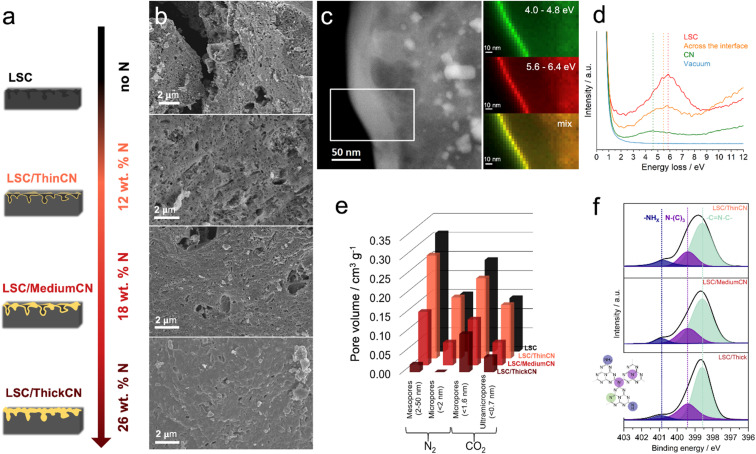
(a) Schematic illustration of the change in the thickness of the CN layer depending on the amount of melamine precursor used; the nitrogen content for each sample obtained from EDX is shown on the right. (b) Secondary electron SEM images of the LSC, LSC/ThinCN, LSC/MediumCN, and LSC/ThickCN samples. (c) ADF-STEM image of the LSC/ThickCN sample. The white rectangle marks the area from which the VEELS spectral data cube was collected. Corresponding spectral imaging maps for different energy windows: 4.0–4.8 eV and 5.6–6.4 eV and mixed signals are shown on the right. (d) Comparison of EELS spectra acquired at the vacuum region, CN, interface, and LSC. (e) Cumulative meso- and micro-pore volumes of materials from N_2_ and CO_2_ physisorption measurements. (f) N1s core levels from XPS and associated chemical structure of CN-cycle.

The final materials are designated as LSC/ThinCN, LSC/MediumCN, and LSC/ThickCN, indicating the increasing thickness of the CN layer, as confirmed by cross-sectional transmission electron microscopy (TEM) images (Fig. S3†). From the annular-dark field scanning transmission electron microscopy (ADF-STEM) images, carbon nitride layers appear brighter compared to LCS. STEM-EDX maps collected from the same spots confirmed that bright areas are CN layers (Fig. S3†). The thicknesses of the CN layers, measured based on the intensity change in the images, were found to be 10 ± 2 nm, 26 ± 6 nm, and 69 ± 14 nm for LSC/ThinCN, LSC/MediumCN, and LSC/ThickCN, respectively. Valence electron energy loss (VEELS) spectral data cubes were collected across the interface of the LSC/ThickCN sample. The VEELS spectra represent the inelastic scattering energy loss of the electron beam by the material's outer shell electrons (Fig. 2c), reflecting the valence electron density. The width of this peak reflects the damping effect of single-electron transitions.^46^ LSC has a peak at 6.0 eV, typical of carbonaceous materials.^47^ The CN coating shows a peak at 4.5 eV, indicating that the electrons are displaced from the outer shell with lower energies compared to the LSC. At the LSC/CN interface (Fig. 2d, orange line), the full width at half maximum of the peak appears much broader compared to LSC and CN. The maximum intensity of this peak is slightly red-shifted, revealing a coupling between the electronic states of CN and LSC. In the vicinity of the interface, the electron beam induces the electron density to flow from the LSC towards the CN, like in the depletion region of a Schottky contact, by reducing the interface energy barrier height. In a similar manner, by inducing the electrons to flow from the LSC to the CN layer, thus generating a negative potential at the CN coating, we expect to enhance the surface adsorption and transport towards the LSC electrode of the sodium ions, eventually improving the electrochemical energy storage performances. It is also worth noticing the components of the electron density are placed outside the material (a few nanometers), *i.e.*, we can depict the evanescent low energy electrons, plasmonically or excitonically bound to the material's surface.

The change in the morphology of the materials with increasing CN layer thickness was investigated using SEM. The thinnest CN coating does not visually affect the carbon surface morphology compared to the bare LSC. By increasing the melamine amount and, accordingly, the CN film thicknesses, the LSC appears to be entirely covered by a CN blanket laying over the carbon structure (Fig. 2b and S4†). In all cases, the EDX elemental maps (Fig. S5†) confirm that the nitrogen is homogeneously distributed on the coated materials without any apparent phase segregation at the micrometer level and with increasing nitrogen content at larger CN film thickness (Table S1 and Supplementary note 2†). The layered structure of the bulk CN was confirmed with XRD, revealing the characteristic peaks of (100) and (002) planes (Fig. S6†).^48^ XRD patterns of LSC/CN also present a (002) peak of CN (Fig. S6 and Supplementary note 3†). No significant difference was observed in the Raman spectra of the materials (Fig. S7†). Thermogravimetric analysis (TGA) (Fig. S8†) in synthetic air and N_2_ shows that the thermal stability of the pristine LSC is significantly improved by the CN coating, confirming the intimate interaction between the materials, resulting in a higher thermal resistance as compared to its single components, which is typical for composite materials (Supplementary note 4†).^26,29^

With regards to the porous structure, the type IV isotherms obtained from N_2_ sorption analysis (Fig. S9†) reveal the mesoporous nature of the materials with multilayer adsorption followed by capillary condensation of nitrogen in the mesopores. According to the density functional theory (DFT) model, pristine LSC has a 498 m^2^ g^−1^ of nitrogen available surface area that is subsequentially reduced down to 4 m^2^ g^−1^ by increasing the thickness of the CN film, blocking the gas access to the porous core of the composite. Such a decrease in the surface area confirms once more the increasing thickness of the CN thin film. The growth of the CN film, and the consequent reduction of nitrogen-available surface area, reduce the surface available for the growth of the natural passivation layer, as its formation during initial charging is usually associated with the electrode's surface area.^49,50^ However, the difference between the cumulative micropore (*d*_pore_ < 2.0 nm) volume of LSC and LSC/ThinCN is observed as marginal (Fig. 2e). To further investigate the potentially available micropores, we performed CO_2_ sorption experiments at room temperature (Fig. S10†),^51^ which reveal a sub-microporous nature of disordered carbon, in good agreement with other hard carbons, however, born at much higher temperatures.^52,53^ Here, the volume of micropores is generally reduced by the CN coating layer. One exception is that the CO_2_-accessible ultramicropore (*d*_pore_ < 0.7 nm) volume of LSC/ThinCN and LSC is similar. However, a further increase in the coating thickness causes an eventual barrier ahead of the ultramicroporous texture, resulting in a significant reduction of the CO_2_-accessible area in both LSC/MediumCN and LSC/ThickCN (Fig. 2e).

The chemical states of the deposited CN film were confirmed with X-ray photoelectron spectroscopy (XPS) and Fourier-transform infrared (FTIR) spectroscopy. In the deconvoluted N1s spectra (Fig. 2f), the peak at 400.9 eV is attributed to the terminal amino groups (–NH_*x*_). In contrast, the peaks at 399.4 eV and 398.6 eV represent the central heptazine-based (N–(C)_3_) and aromatic nitrogen (C=N–C), respectively.^54–56^ Here, the areal ratio of the aromatic to central nitrogen remains constant, indicating that the film retains its structure without increasing the terminal edge groups associated with the defect sites. Additionally, we can further confirm that pristine LSC does not contain any nitrogen before CN deposition (Fig. S11c and Table S1†). In terms of heteroatoms, the pristine LSC contains sulfur features as –C–SO_*x*_ and –C–S– states (Fig. S11b†). From the C1s spectra (Fig. S12†), the peak at 284.6 eV is attributed to the C–C bonds, whereas the peaks at 286.4 eV and 288.3 eV to the carbon bond to amino-terminal groups and heptazine rings (–N=C–N–), respectively.^31^ The chemical structure of the films was further investigated with FTIR (Fig. S13†). Typical CN-heterocycle heptazine breathing mode (810 cm^−1^) and CN-cycle vibrations (1258 cm^−1^ to 1630 cm^−1^) peaks match with previous reports on CN materials.^57,58^

**Fig. 3:**
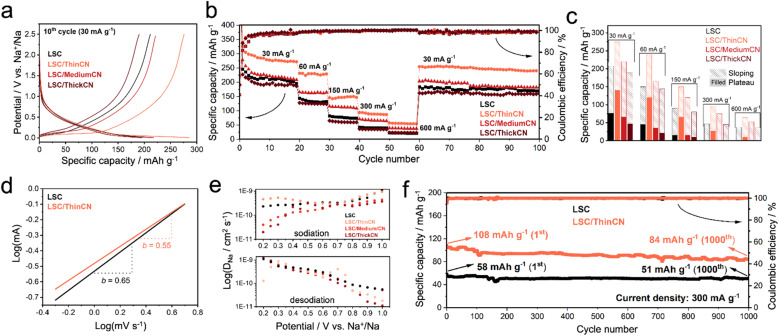
Electrochemical performance of LSC, LSC/ThinCN, LSC/MediumCN, and LSC/ThickCN in half-cell SIB. (a) 10th GCD curves of half-cells at 30 mA g^−1^. (b) Rate-capability performance of the materials. (c) Stacked column chart of materials at different current densities with the distribution of the plateau and sloping capacities. (d) log(peak anodic current) *vs.* log(scan rate) plots of LSC and LSC/ThinCN from CV. (e) Diffusion coefficients of materials from GITT with respect to sodiation and desodiation potentials. (f) 1000 cycle stability tests of LSC and LSC/ThinCN at a constant current density (300 mA g^−1^).

Before galvanostatic charge and discharge (GCD) measurements, electrodes were tested with electrochemical impedance spectroscopy (EIS) to evaluate the overall electrode resistance and resistance contribution from CN coating (Fig. S14†). From the relative Randles circuit model, the difference in equivalent series resistance (ESR, a combination of bulk electrolyte and electrode intrinsic resistances) of the electrodes with different CN thicknesses was marginal (*R*_2_, Table S2†). However, the growth of a 69 nm-thick CN layer significantly increases the charge transfer resistance from 86.8 Ω cm^−2^ to 396.5 Ω cm^−2^ due to the electrically insulating characteristics of the CN layer.^26^ The GCD curves reveal that the CN coating increases the initial coulombic efficiency (ICE) of the active electrode material, from 25% for pristine LSC to 50% for LSC coated with CN (Fig. S15†). This improvement was not further optimized but just supports our concept that a stable CN coating reduces the electrode's surface area available for the electrolyte and thus suppresses the growth of the natural passivation layer.^59^ Following the stable formation of the natural SEI layer, LSC, LSC/ThinCN, LSC/MediumCN, and LSC/ThickCN provide reversible capacities of 214 mA h g^−1^, 275 mA h g^−1^, 220 mA h g^−1^, and 190 mA h g^−1^ at 30 mA g^−1^ (10th cycle), respectively, with coulombic efficiencies, in all cases, over 99% (Fig. 3a). The deposition of a 10 nm thick and homogeneous CN film improves not only the ICE but also has a remarkable effect on sodium storage capacity, increasing it by more than 30%. Such a striking improvement is indicative of an electronic impact of the artificial passivation layer on the carbon storage material, especially on the non-capacitive faradaic storage mode. By means of the melamine amount inserted in the CVD setup, we could provide a unique thickness control by process, achieving a significant improvement in the battery performance. The materials' structure and electronic/chemical interactions between the CN film create an interface layer that improves the sodium storage performance. As we know from similar multilayer constructions,^30^ the electrochemically rather reductive carbon transfers the electrons rapidly to the CN with its rather positive work function, creating a Schottky transfer layer.^60^ This negatively doped CN at a lower thickness of *ca.* 10 nm (comparable to the Schottky length) possesses the lowest charge transfer resistivity among our CN-coated electrodes. The electron-rich, negatively charged CN would then attract the sodium ions through its pores to the carbon electrode. There, the sodium is reduced to Na(0) in the plateau region, and this electron-rich sodium back-donates electron density to the carbon phase, which gives its electron to the carbon nitride, closing the cycle. This polarization-backdonation-stabilization effect explains the broader plateau with negligible change in the capacitive range. However, increasing the thickness, this effect is reduced and eventually inverted, both by the kinetics and voltage loss due to charge transfer resistivity. It is worth noting that the improvement in electrochemical performance is not attributed to the vacuum thermal treatment during the CVD process or the contributions from the CN. For example, the capacity of the pristine LSC treated in the CVD tube in the absence of the melamine decreases significantly, and pure CN does not provide sodium storage capacity when tested in identical conditions (Fig. S16 and Supplementary note 5†).

At higher current densities, where the electrodes mostly rely on the pseudo-capacitive storage mode, the rate capability of the pristine LSC is also improved by CN coating (Fig. 3b and c). Exemplarily, the specific capacity of pristine LSC is doubled with the LSC/ThinCN when charging at 300 mA g^−1^ and 600 mA g^−1^. Even though the pristine LSC and the LSC/MediumCN provide similar storage capacities at 30 mA h g^−1^, LSC/MediumCN retains more capacity at high current densities, indicating that the CN film significantly improves the rate capability also. According to the intercalation/filling-based model, high potential plateau capacity is due to the intercalation of sodium ions within planes and micropores present in the disordered carbons, which occurs when the low-energy supercapacitive adsorption is saturated.^11,61,62^ Here, the effect of CN film on the supercapacitive mode (slope region of GCD curve) is negligible as the electrodes give almost identical sloping behavior. In contrast, a major change in the performance, mainly in the plateau region, is observed at around 0.1 V (*vs.* Na^+^/Na). The plateau capacity of pristine LSC is extended from 80 mA h g^−1^ (a total of 214 mA h g^−1^) to 140 mA h g^−1^ (a total of 275 mA h g^−1^). The change in the electrochemical storage mechanism between LSC and LSC/ThinCN is clarified through cyclic voltammetry (CV) at different scan rates (Fig. S17†).^63^ The slope of the log(*i*, peak anodic current) *vs.* log(*ν*, scan rate) plot provides a kinetic analysis of the sodium storage mechanism. When the slope (*b*-value) is close to 1, the electrochemical process is considered convective and thereby related to surface processes. In contrast, a bulk-diffusion-controlled mechanism shows a *b*-value close to 0.5.^64^ Here, the corresponding *b*-values (Fig. 3d) are calculated to be 0.65 and 0.55 for LSC and LSC/ThinCN, respectively, in good agreement with the assignment that the extended plateau region of the LSC/ThinCN is associated with the diffusion-controlled insertion of sodium ions. Apparent sodium-ion diffusion coefficients of the materials were calculated from the galvanostatic intermittent titration technique (GITT) following the procedure in Supplementary note 6.† As shown in Fig. 3e, diffusion coefficients of materials vary in the order of 10^−9^ and 10^−11^ cm^2^ s^−1^ depending on the sodiation and desodiation potential. During the sodiation, diffusion coefficients of the LSC/MediumCN and LSC/ThickCN were lower than the LSC and LSC/ThinCN. However, during the desodiation, a sharp decrease in the diffusion coefficient of the LSC/ThinCN is observed through the plateau around 0.3 V (*vs.* Na^+^/Na) (Fig. S18†), already observed in GITT experiments of high-capacity hard carbons.^65^ This phenomenon was previously reported and attributed to the presence of some electrochemical reactions^66^ (*e.g.*, Na^0^ to Na^+^), being special for only LSC/ThinCN.

We assess that the CN coating, in this case, does not act “only” as artificial interphase, matching the three principles of mechanical stability, ionic conductivity, and chemical passivation^30,31,67^ but it also takes a key role in the reduction potential of the sodium ion exploiting the heterojunction effect at the interface, as explained above. By charge transfer from carbon nitride to the sulfur-doped carbon, more sodium sites are now accessible in underpotential mode. For these reasons, we would address this phenomenon by referring to this type of layer as an “inter-active interphase”. The more efficient storage of sodium ions with a large fraction of capacity achieved around 0.1 V (*vs.* Na^+^/Na) reduces the nominal anodic potential, thus improving the expected energy density in the full-cell configuration.

To test the long-term stability, LSC and LSC/ThinCN were cycled 1000 times at 300 mA g^−1^ (Fig. 3f). The capacity retention of 88% and 78% is observed for LSC and LSC/ThinCN, respectively. The higher stability of the pristine LSC electrode is associated with the dominant pseudo-capacitive adsorption of sodium ions, which is less harmful to the electrode structure as compared to the slow ion insertion process that causes local disturbance of graphitic-like domains in carbons.^68^ Still, considering the improvement in specific capacity for LSC/ThinCN (+65% at the 1000th cycle at 300 mA h g^−1^), the stability improvement is at least not obvious.

**Fig. 4:**
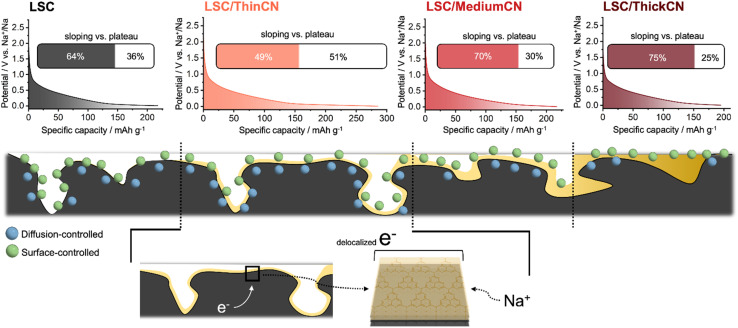
The contribution of sloping and plateau capacities based on the sodiation curves at 30 mA g^−1^. Illustration of sodium accumulation at the interface.

Thereby, the conformal CN coating is a critical driver for improving the electrochemical properties of the carbon derived from a sustainable source, and the film thickness is assessed to be the most crucial parameter to boost sodium storage, especially in the non-capacitive faradaic storage mode. The main reason for this was attributed to the heterojunction effect between CN and the LSC. As we already know, the carbon derived from lignosulfonate is not a noble carbon but rather a reductive carbon,^69,70^ indicating that its Fermi level is much higher than the one of the artificial-SEI.^31,69^ Here, LSC donates electrons to the CN that subsequently accumulates sodium ions due to the polarization effect (Fig. 4). The positive polarization of the LSC simplifies the integration of the highly electron-rich Na(0), thus recreating a compensating polarization-backdonation-stabilization effect enhancing the sodium storage at the composite electrode. Charge transfer and the coupled increase of polarity also tune the pathways to the microporous structure already available in the material, allowing smooth transfer of ions *via* improved wettability and adsorption enthalpy, also improving the intercalation. Increasing the CN layer thickness too much, however, increases the diffusion resistivity and shifts the dominant storage mechanism from diffusion-controlled to surface-controlled. As highlighted in Fig. 4, the share of a plateau capacity of pristine LSC is improved from 36% to 51% with a thin CN coating, whereas further deposition decreases its contribution to only 25%.

## 3. Conclusion

Due to the unique electrochemical compatibility between the host carbon substrate and CN artificial interphase, composite material offers remarkable improvements in the electrochemical performance in terms of ICE, rate capability, and capacity with an optimum CN thickness. More importantly, the plateau capacity of the pristine electrode nearly doubles, pointing to a significant increase in energy density. The combination between a reductive carbon and a thickness-controlled CN film deposition successfully enables the exploitation of the heterojunction effect at the materials' interfaces, especially for the thinnest coating. Further increase in the conformal film thickness does not improve the total specific capacity and diffusion-controlled sodium storage but slightly enhances the rate-capability compared to the pristine LSC. In particular, we demonstrated that the CN thin film is a promising candidate as artificial interphase to improve the sodium storage performance of carbonaceous anode materials, even applicable to the undemanding materials derived from different sources such as biowastes.

## Conflicts of interest

There are no conflicts to declare.

## Supplementary Material

TA-011-D2TA07391A-s001
