# Supplementary material for: Conformal carbon nitride thin film inter-active interphase heterojunction with sustainable carbon enhancing sodium storage performance
Source: J Mater Chem A Mater. 2022 Dec 21;11(3):1439–46. doi: 10.1039/d2ta07391a (PMC9844057; doi:10.1039/d2ta07391a)
Supplement: TA-011-D2TA07391A-s001 [file TA-011-D2TA07391A-s001.pdf]

Electronic Supplementary Material (ESI) for Journal of Materials Chemistry A.
This journal is © The Royal Society of Chemistry 2022

Supporting Information

Conformal carbon nitride thin film inter-active interphase heterojunction with sustainable carbon enhancing sodium storage performance

Enis Oğuzhan Eren,[a] Evgeny Senokos,[a] Zihan Song,[a] Elif Begüm Yılmaz,[a] Irina Shekova,[a] Bolortuya Badamdorj,[a] Iver Lauermann,[b] Nadezda V. Tarakina,[a] Majd Al-Naji,[c] Markus Antonietti,[a] and Paolo Giusto[a]*

[a] Department of Colloid Chemistry, Max Planck Institute of Colloids and Interfaces, Potsdam 14476, Germany

[b] PVcomB, Helmholtz-Zentrum Berlin für Materialien und Energie, Berlin 12489, Germany

[c] Technische Universität Berlin, Berlin 10623, Germany

**E-Mail: paolo.giusto@mpikg.mpg.de*

Table of Contents

Experimental section 3
Synthesis of disordered carbon from lignosulfonate 3
Applying carbon nitride (CN) thin films by chemical vapor deposition (CVD) method 3
Electrode preparation and electrochemical measurements 4
Physico-chemical characterizations 5
Figure S1 6
Figure S2 7
Supplementary note 1 7
Figure S3 8
Figure S4 9
Figure S5 10
Table S1 10
Supplementary note 2 10
Figure S6 11
Supplementary note 3 11
Figure S7 12
Figure S8 13
Supplementary note 4 13
Figure S9 14
Figure S10 15
Figure S11 16
Figure S12 17
Figure S13 18
Figure S14 19
Table S2 19
Figure S15 20
Figure S16 21
Supplementary note 5 21
Figure S17 22
Figure S18 23
Supplementary note 6 24
Figure S19 25
Supplementary references 26

Experimental section

Synthesis of disordered carbon from lignosulfonate. A sodium lignosulfonate (58 wt. %, Domsjö Fabriker) as a carbon source; urea (4 wt. %, Sigma-Aldrich) and D-glucose anhydrous (4 wt. %, Sigma-Aldrich) as a binder mixture; DI water (10 wt. %); ZnO nanoparticles (24 wt. %, NanoAmor, 20 nm) as porogen were mixed using a commercial kitchen kneader (Bosch, Germany).[1, 2] DI water in drops was slowly added to the mixture as a plasticizer to provide consistency for extrusion. Finally, the low-moisture mixture was extruded and cut in the form of pellets (1 mm in diameter and 1.5 mm in length) using a commercial noodle extruding machine (La Monferrina, Italy) (Figure S1a) following the procedure developed by Brandi et al.[1] The role of glucose/urea binder is to provide crosslinking while carbonizing the formed pellets and generating an intact rigid sulfur-rich carbon. In the absence of glucose/urea binder, the physical mixture will lead to random evaporation of Zn and will not result in the desired interconnected hierarchical material. The extruded pellets were dried at room temperature for 12 hours. The dried pellets were put in an ashing furnace (Nabertherm, Germany), heated to 120 ºC with a heating rate of 3 K min^{-1}, and kept for 2 hours to crosslink under N_2 atmosphere. Subsequently, the oven was heated to 950 ºC with a heating rate of 3 K min^{-1} and kept at this temperature for 2 hours to finalize step-wise condensation. At this stage, carbothermal reduction of ZnO nanoparticles to Zn metal occurs, and the Zn metal leaves the framework. In the end, the carbonization yield was measured as 30%. Carbonized pellets (Figure S1b) were ground to powder and washed with 0.1 M HCl solution under continuous stirring to remove the residual Zn (Supplementary note 1).

Applying carbon nitride (CN) thin films by chemical vapor deposition (CVD) method. The procedure developed by Giusto et al.[3] was followed as a coating model. PlanarGROW-3S-OS (UK) CVD system with a 3-in. quartz tube was used for the deposition of CN films on LSC. In general, LSC powder (150 mg) was placed horizontally in the center of the second chamber, and a glass boat containing melamine

(99%, Sigma-Aldrich) precursor (1 g for LSC/ThinCN, 2 g for LSC/MediumCN, and 5 g for LSC/ThickCN) in the center of the first chamber. The vacuum was pulled down to 10 Torr, and the temperature at the substrate was raised to 550 °C, with 50 sccm nitrogen flow as carrier gas. As soon as the substrate was at 550 °C, the melamine was heated up to 300 °C at a rate of 10 K min^{-1} and kept for an additional 30 min. The samples were cooled to room temperature naturally. The color of samples changes significantly from black to bluish depending on the precursor amount (Figure S1c).

Electrode preparation and electrochemical measurements. Electrodes were fabricated by mixing LSC, LSC/ThinCN, LSC/MediumCN, and LSC/ThickCN with the conductive carbon black (Super-P, Alfa Aesar) and polyvinylidene difluoride (PVDF, Kynar HSV-900) binding agent at a weight ratio of 8:1:1. PVDF was dissolved in N-Methylpyrrolidone (NMP, Sigma-Aldrich). The slurry was cast on an aluminum foil (15 μm) using an automatic doctor blade film applicator (mtv messtechnik, Germany) and dried overnight in the vacuum oven (Thermo Fisher, USA) at 80 °C. The final loading of the active materials was measured as ~1.0 mg cm^{-2} for all samples. All electrochemical measurements were conducted using two-electrode Swagelok-type cells on a Biologic MPG-2 instrument (France). Swagelok-type cells are assembled in an argon-filled glovebox (MBRAUN, Germany) with an H_2O and O_2 content of less than 0.1 ppm. 1M $NaPF_6$ in ethylene carbonate (EC)/ethyl methyl carbonate (EMC) (3:7 in vol., E-Lyte GmbH, 200 μL) was used as an electrolyte, and glass fibers (Whatman GF/C) served as the separators, and a thin piece of sodium metal (99.5%, Sigma-Aldrich) was used as both counter and reference electrode. For the reproducibility of the data, at least four Swagelok-type cells were prepared for each sample. The galvanostatic charge-discharge curves of the half-cells were collected in the potential range of 0.005-2.5 V (vs. Na^+/Na). Before the measurements, half-cells were rested for three hours. Cyclic voltammetry (CV) measurements were carried out at scan rates of 0.5, 1.0, 2.0, and 5.0 mV s^{-1} in the potential range of 0.01-2.0 V (vs. Na^+/Na). Electrochemical impedance spectroscopy (EIS) was performed with an AC

perturbation of 10 mV in the frequency range of 10^{-1} to $2 \cdot 10^4$ Hz. All electrochemical measurements were conducted at room temperature. Galvanostatic intermittent titration technique (GITT) was used to calculate the sodium-ion diffusion coefficients of the electrodes. During the experiments, current pulses (30 mA g^{-1}) were applied for 600 s, and relaxation potentials were measured for 3600 s. The diffusion coefficients were calculated according to Supplementary note 6.

Physico-chemical characterizations. X-ray photoelectron spectroscopy (XPS) was performed using the CISSY equipment (Helmholtz-Zentrum Berlin, Germany) with a SPECS XR 50 X-ray source using the Mg K_α radiation. Photoelectrons were analyzed using a hemispherical analyzer (CLAM4 by VG). Binding energy calibration was performed using clean samples of gold, copper, and silver foil. Indium foil (99.99%, Sigma-Aldrich) was used as a substrate. The Shirley-type background and Lorentzian-Gaussian (mixed) models were used for the fittings. The crystallinity of the material was determined by X-ray diffraction (XRD) using Rigaku SmartLab (Japan, Cu K_α, 0.154 nm). Raman spectroscopy was obtained using WITec Alpha 300 (Germany) confocal Raman microscope with an excitation wavelength of 532 nm. Thermo Scientific Nicolet iD7 (USA) spectrometer was used as a Fourier-transform infrared (FTIR) spectrometer. Thermogravimetric analysis (TGA) was conducted using NETZSCH TG-209 Libra (Germany) under a synthetic air and N_2 atmosphere at a heating rate of 10 K min^{-1}. Physisorption measurements were performed on a Quantachrome Quadrasorb SI (Austria) at 77 K for N_2 and 273 K for CO_2. Samples were degassed overnight before the measurements. The density functional theory (DFT) method was used to evaluate the pore size distribution (PSD) of the materials employing adsorption isotherms. Inductively coupled plasma optical emission spectrometry (ICP-OES) was conducted with PerkinElmer Optima 8000 (USA). Scanning Electron Microscopy (SEM) imaging was performed using the Zeiss LEO 1550-Gemini (Germany) system with acceleration voltages of 3, 5, and 10 kV. An Oxford Instruments X-MAX (UK) 80 mm^2 detector was used to collect the energy-dispersive X-ray (EDX) data.

For scanning transmission electron microscopy (STEM) observations, samples were embedded in Spurr resin (Electron Microscopy Sciences) and polymerized at 65 °C. Ultrathin sections (80-100 nm thick) were obtained from trimmed resin blocks by ultramicrotomy (Leica Ultracut UCT, Germany), using an Ultra 35° diamond knife (Diatome). The first cuts were routinely discarded, ensuring fresh, non-damaged surfaces. The slices were floated on water, and deposited on holey carbon coated Cu grids. The STEM study was performed using a double Cs corrected JEOL JEM-ARM200F (Japan) (S)TEM operated at 80kV and equipped with a cold-field emission gun and high-angle silicon drift Energy Dispersive X-ray (EDX) detector (Jeol JED-2300 100 mm^2, Japan) (solid angle up to 0.98 steradians with a detection area of 100 mm^2). Annular Dark Field Scanning Transmission Electron Microscopy (ADF – STEM) images were collected at a probe convergence semi-angle of 25 mrad. The so-called "beam shower" procedure was applied for 30 minutes; it was necessary for reducing hydrocarbon contamination during subsequent imaging at high magnification. Images were analyzed with Gatan's microscopy suite (GMS) version 3.4.

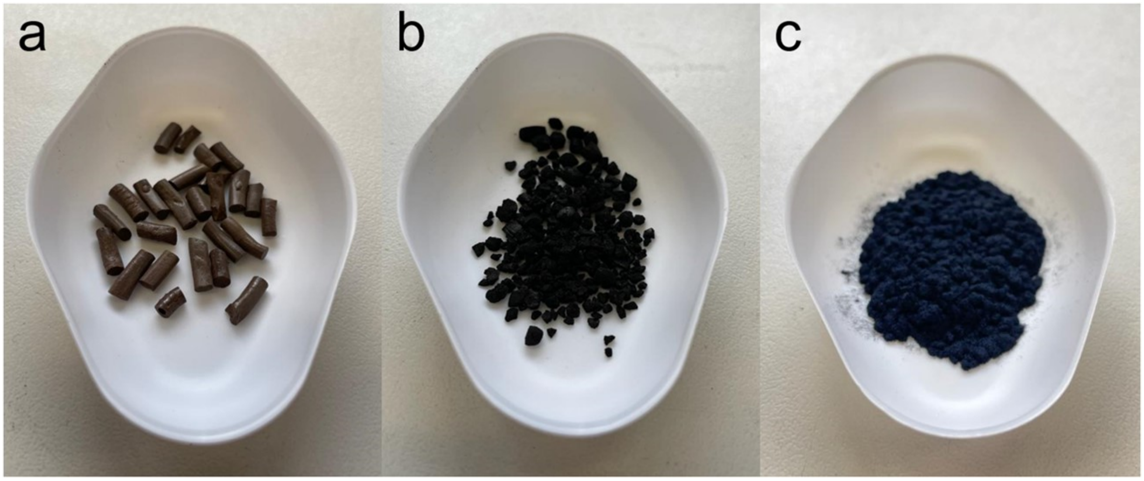

Figure S1. (a) Pellets after extrusion. (b) Pellets after pyrolysis (LSC). (c) LSC after CN deposition (LSC/ThickCN) with a dark bluish appearance.

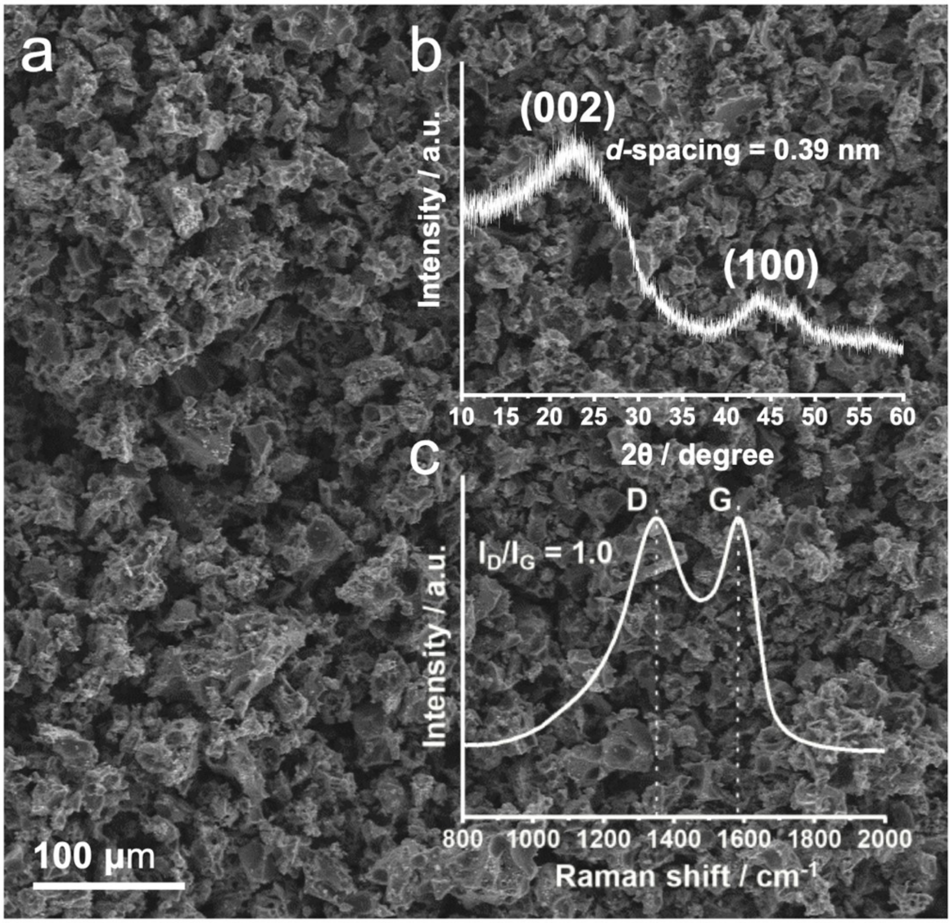

Figure S2. (a) SEM image of the final LSC powder. (b) XRD pattern and calculated *d*-spacing from Bragg's law. (c) Raman spectrum with I_D/I_G peak intensity ratio (fitted with Gaussian and Lorentzian models).

Supplementary note 1. XRD pattern also presents four weak features at 28.5° (111), 32.5° (200), 47.3° (220), and 56.5° (311) that are attributed to the formation of ZnS[4] that is unavoidable during the condensation of lignosulfonate. ICP-OES confirms the presence of residual Zn in the framework despite washing with different chemicals, e.g., HCl (1.9 wt. % Zn), EDTA (3.0 wt. % Zn), and NH_3Cl (7.0 wt. % Zn). After comparing the post-treatments, washing with HCl was chosen as a primary method for LSC. A trace amount of residual Na (<1.0 wt. %) from sodium lignosulfonate is also confirmed.

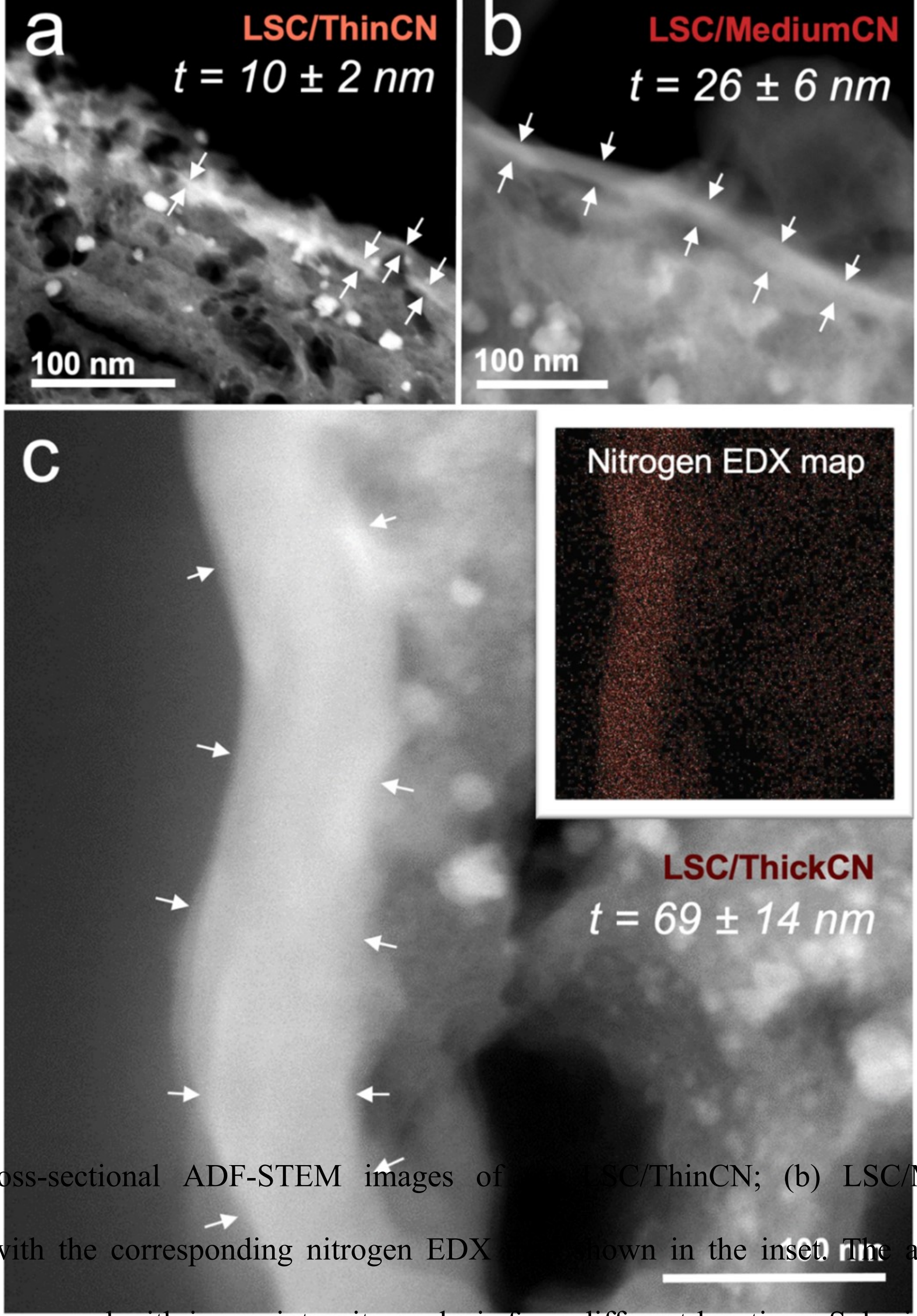

Figure S3. Cross-sectional ADF-STEM images of [illegible]SC/ThinCN; (b) LSC/MediumCN; (c) LSC/ThickCN with the corresponding nitrogen EDX [illegible]hown in the inset. The average CN film thicknesses are measured with image intensity analysis from different locations. Spherical nanoparticles in carbon scaffold are confirmed as ZnS.

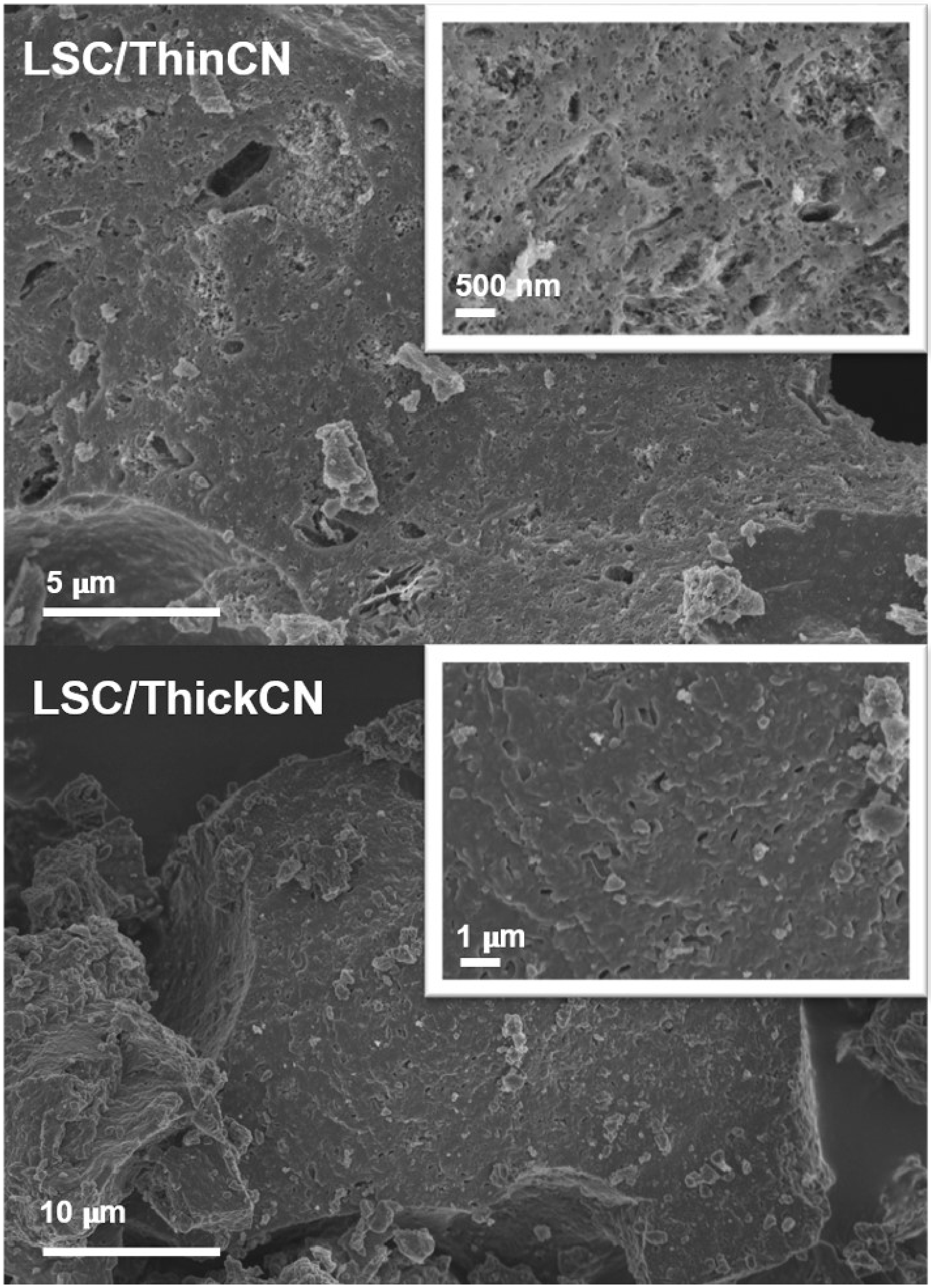

Figure S4. Low and high-magnification SEM images of the thin and thick CN reveal the dense coating of a highly porous and rough surface.

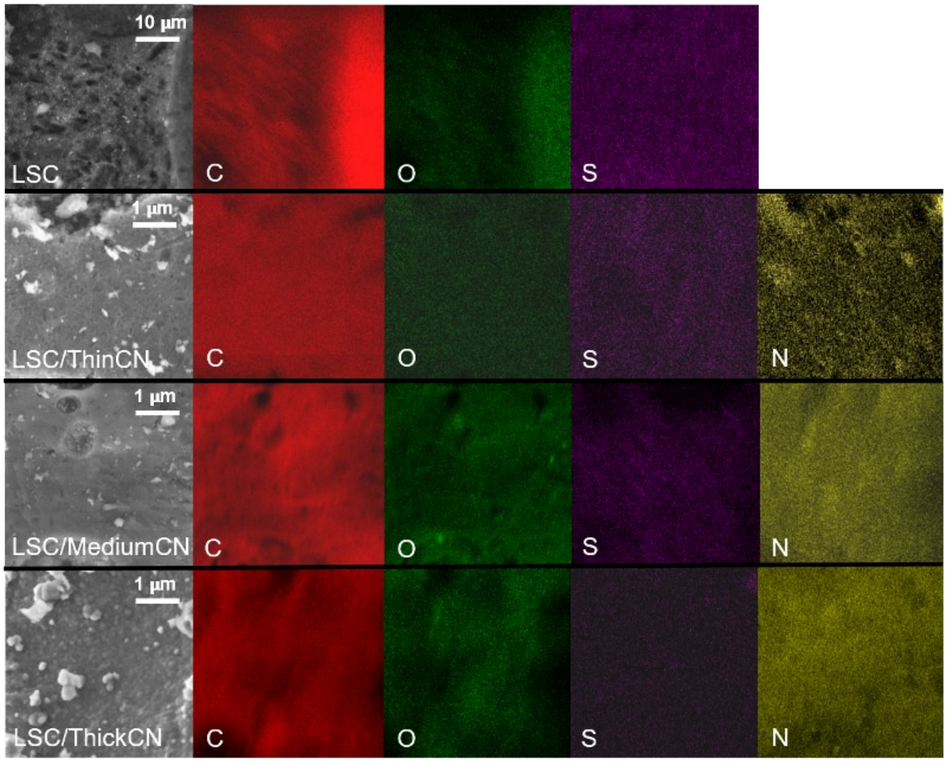

Figure S5. C, O, S, and N elemental mappings from EDX show homogeneous distribution of elements along the framework without significant localizations.

Table S1. Elemental compositions (wt. %) from EDX analysis.

**Sample**	**C**	**O**	**N**	**S**	**Zn/Na**
LSC	87.3 ± 2.7	4.4 ± 0.6	n/a	4.2 ± 1.5	4.1 ± 1.1
LSC/ThinCN	82.7 ± 3.7	1.7 ± 0.5	11.6 ± 2.8	1.3 ± 1.2	2.7 ± 0.6
LSC/MediumCN	71.5 ± 3.0	3.7 ± 0.3	17.7 ± 3.4	2.6 ± 1.1	4.5 ± 1.2
LSC/ThickCN	63.1 ± 3.2	3.6 ± 1.0	25.9 ± 3.3	3.0 ± 0.5	4.4 ± 3.4

Supplementary note 2. The mean and the standard deviation of the elemental compositions are calculated based on at least five spots in each sample. A negligible amount of Cl (due to HCl) was found in some spots that were not included in the statistical analysis. The nitrogen compositions from EDX are primarily attributed to the CN film due to the typical depth resolution of the EDX (usually around 100 nm),[5] resulting in the scattering from the CN being more intense than the bulk material.

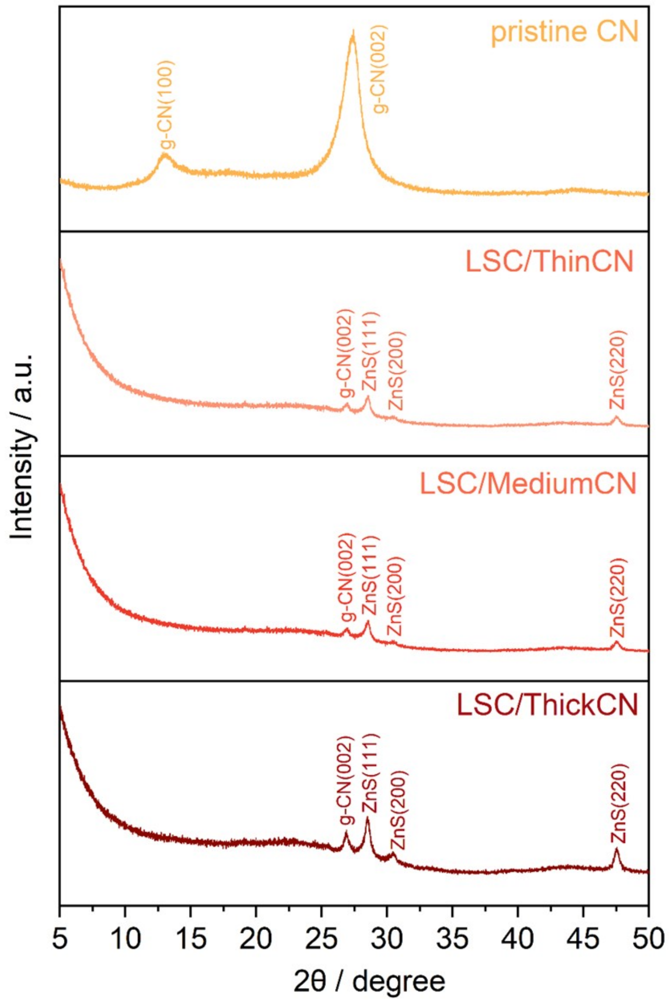

Figure S6. XRD patterns of the materials, including pure CN (polymerized at the same thermal conditions).

Supplementary note 3. XRD of Pristine CN reveals two characteristic peaks at 13.0° (100) and 27.5° (002) of the CN, confirming its layered structure.[6] After deposition on the LSC, the (002) peak of the CN is preserved in the diffraction patterns. Furthermore, it is also observed that peaks from ZnS (See supplementary note 1) become more pronounced due to its recrystallization after CN deposition.

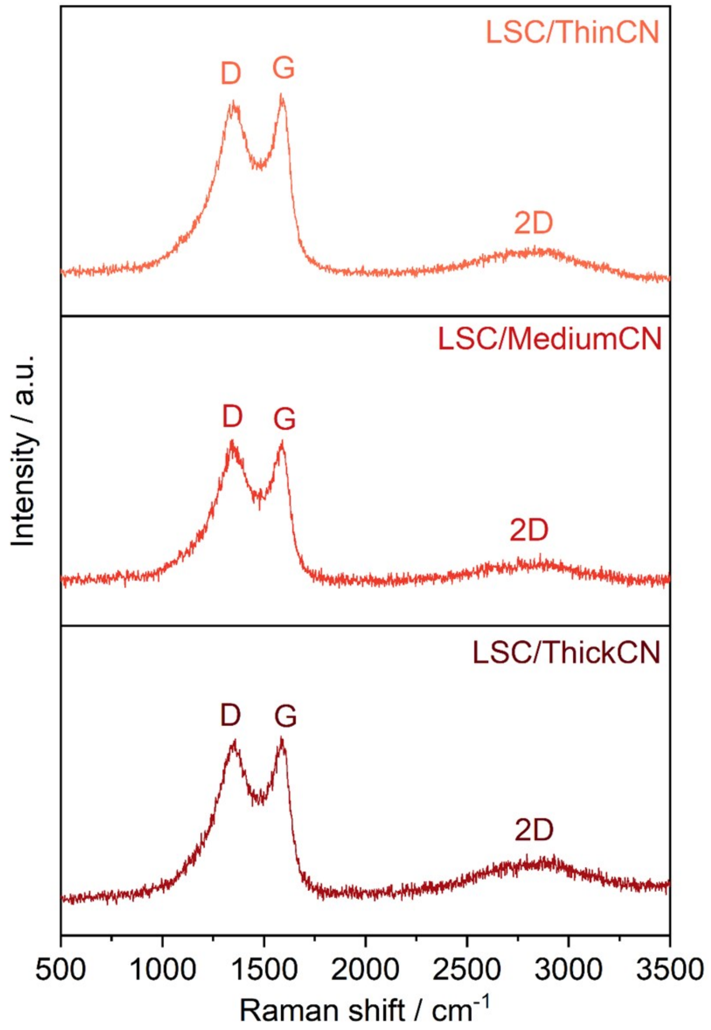

Figure S7. Raman spectra of the LSCs with different CN thicknesses. All materials and the pristine LSC (Figure S1c) have an almost identical *D* to *G* peak intensity ratio associated with the sp^2 hybridization and the oscillations of the breathing mode.

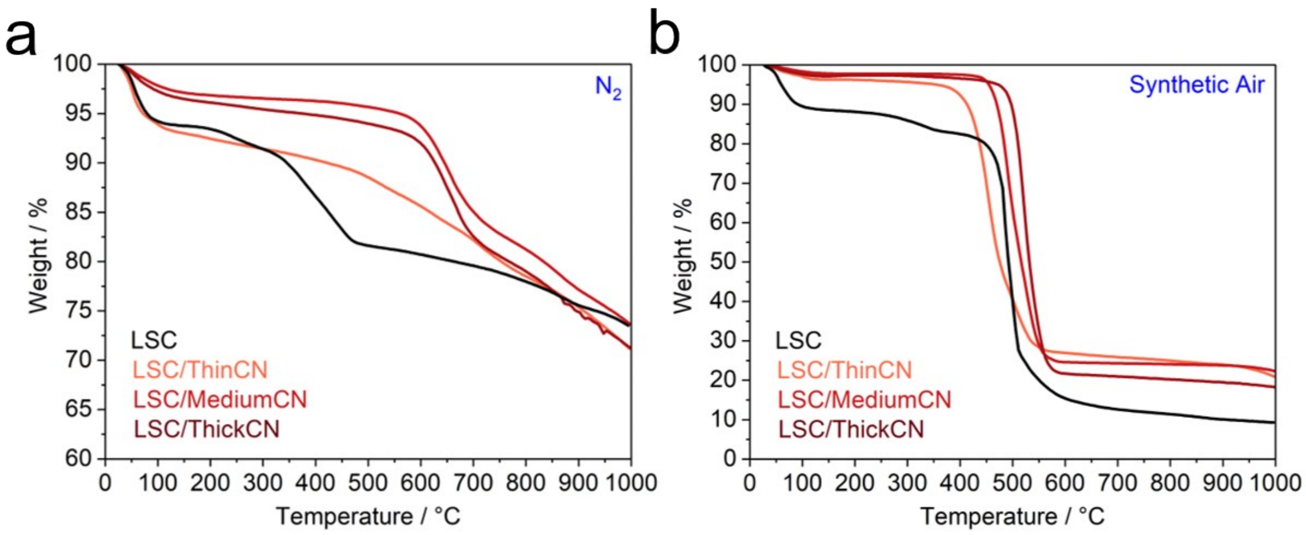

Figure S8. TGA profiles of the materials under (a) N_2 and (b) synthetic air atmosphere reveal an increase in thermal stability.

Supplementary note 4. It is worth underlining that the composite material has a considerably higher thermal stability in the air, with ca. 24% leftover at 900°C for the LSC/ThinCN material, with respect to only 10% leftover for the pristine LSC carbon (Figure S6). This is especially remarkable if we consider that the CN thin films are quantitatively decomposed at 650°C in air.[7] This is typical for composite materials with intimate contact, resulting in a higher thermal resistance than its single counterparts.[7, 8]

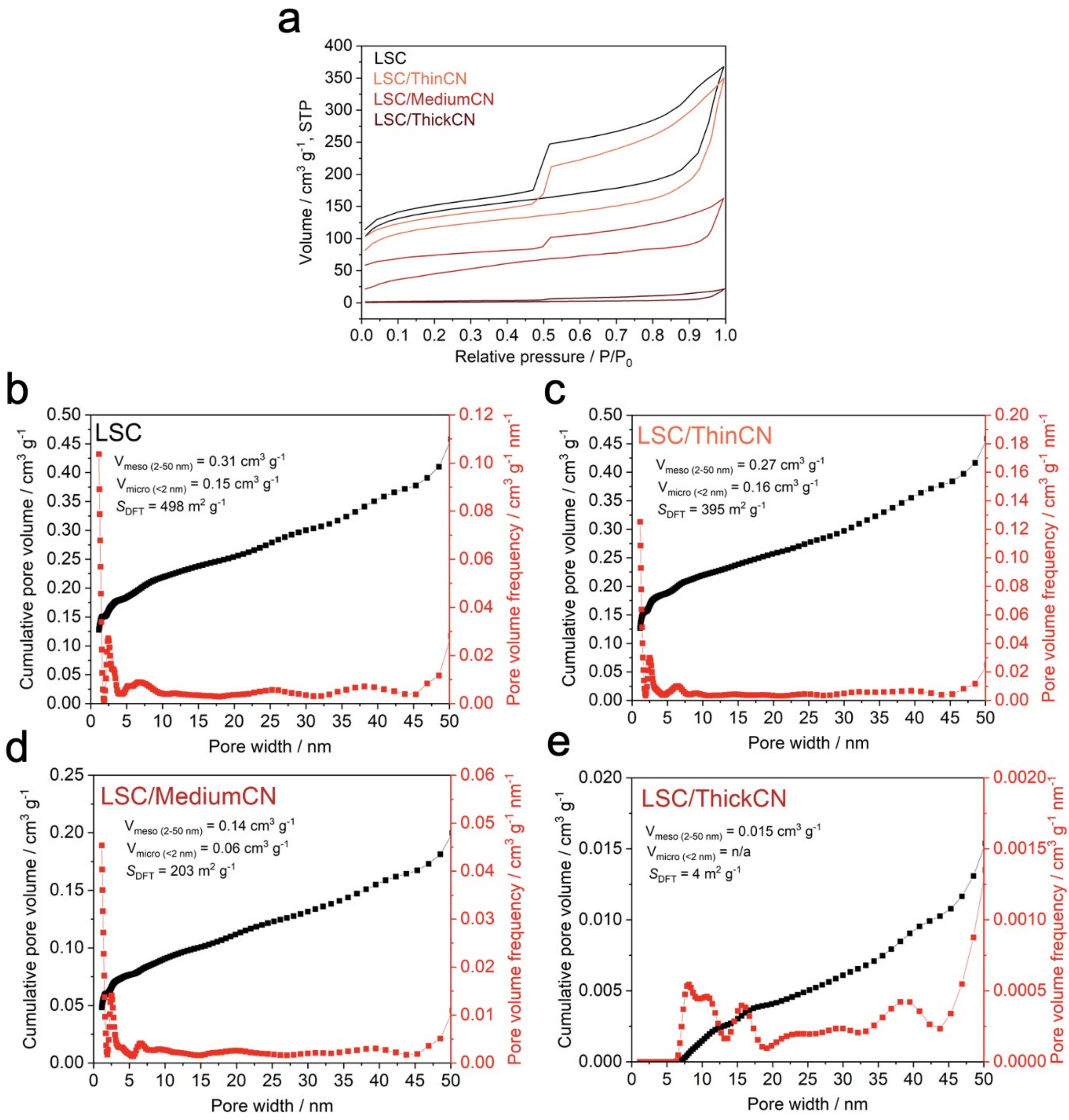

Figure S9. (a) Nitrogen adsorption and desorption isotherms (Type IV) of materials. Pore size distribution (PSD) and cumulative pore volume graphs of (b) LSC, (c) LSC/ThinCN, (d) LSC/MediumCN, and (e) LSC/ThickCN.

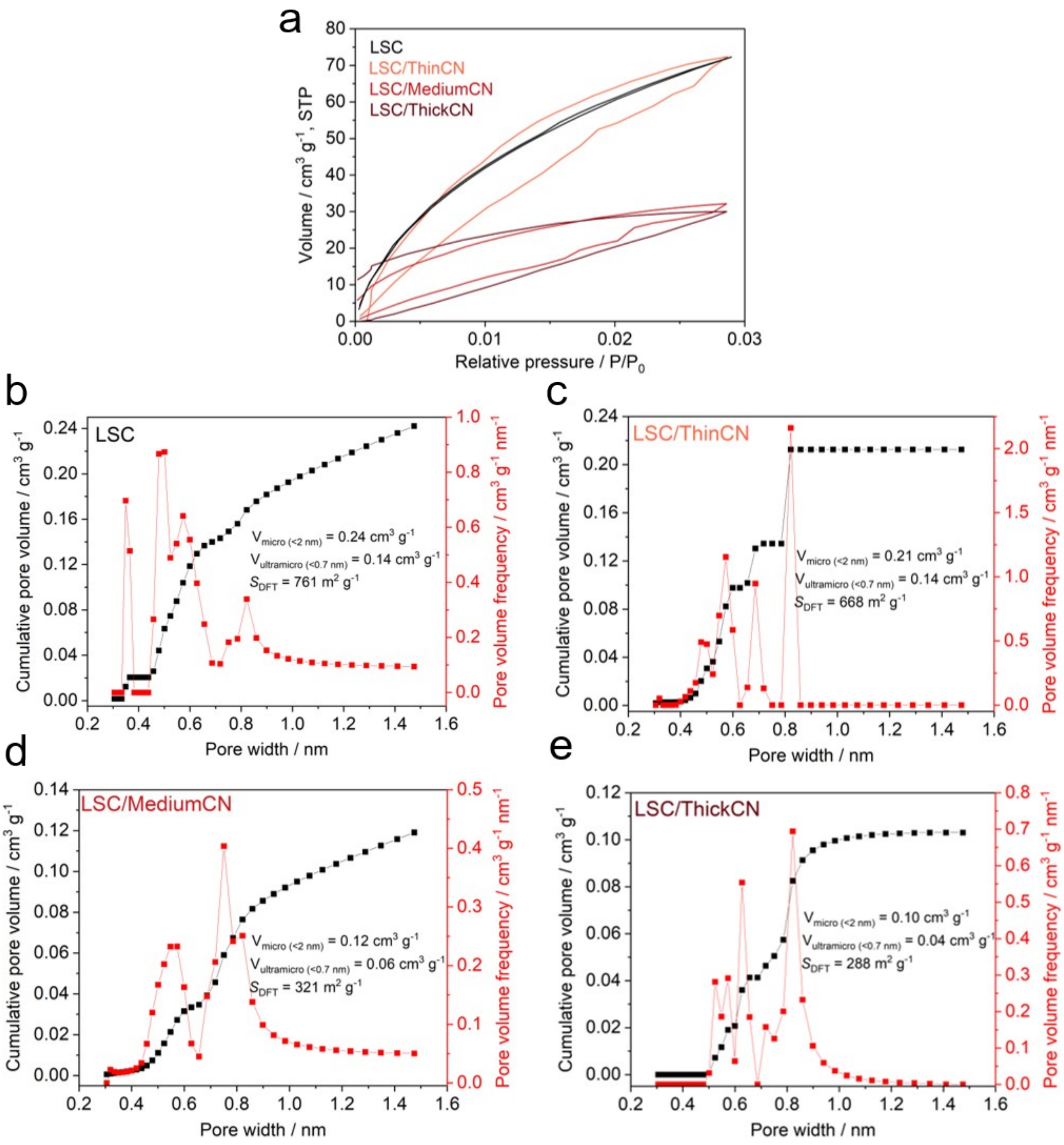

Figure S10. (a) CO_2 adsorption and desorption isotherms of materials. PSD and cumulative pore volume graphs of (b) LSC, (c) LSC/ThinCN, (d) LSC/MediumCN, and (e) LSC/ThickCN.

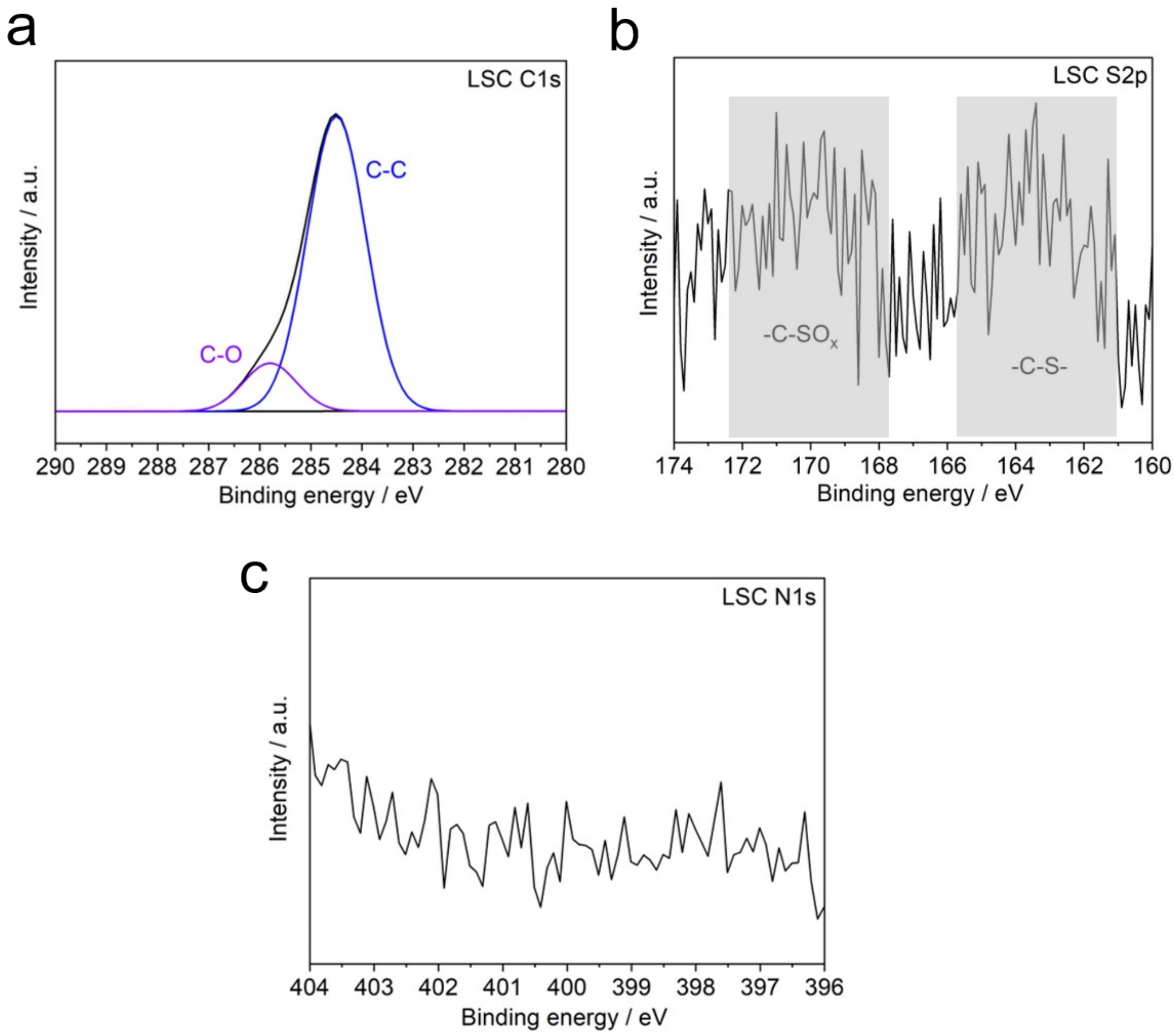

Figure S11. XPS spectra of the pristine LSC. (a) C1s core-level with C–C and C–O peaks. (b) S2p core-level. Sulfur is present due to the nature of the lignosulfonate. The features positioned around 170 eV refer to the –C–SO_x units, whereas the ones around 164 eV are due to the thiophene-type covalent –C–S– bonds.[9-11] The sulfur content in the LSC was estimated at around 4.1 wt. % via XPS, which of approximately 2 wt. % belongs to the ZnS in the scaffold. (c) N1s core-level confirms that LSC has no nitrogen contribution before CN deposition.

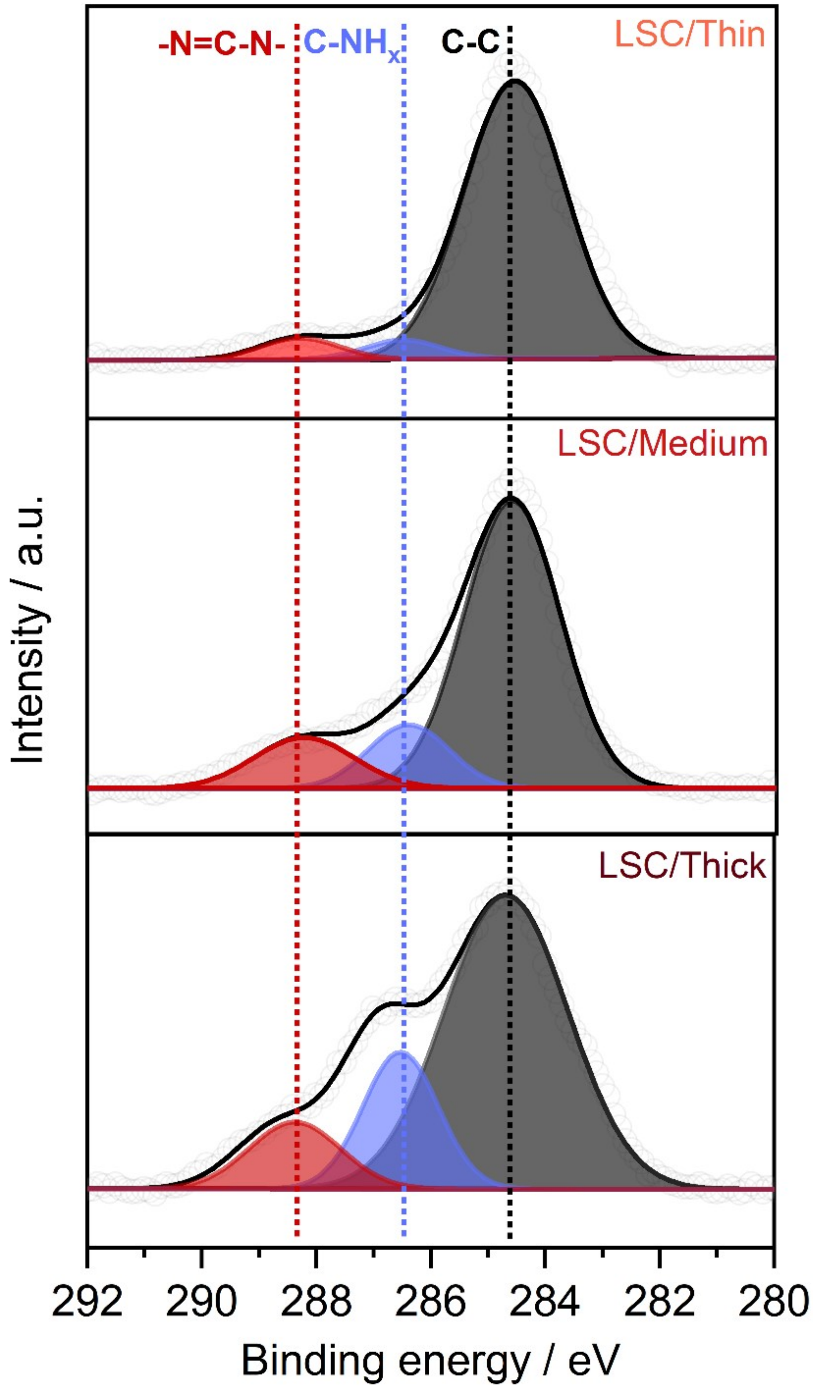

Figure S12. C1s core levels of LSC/ThinCN, LSC/MediumCN, and LSC/ThickCN. Increasing the coating thickness decreases the signal from pristine LSC, resulting significant increase in C–NH_x and CN_2 to C–C ratio.

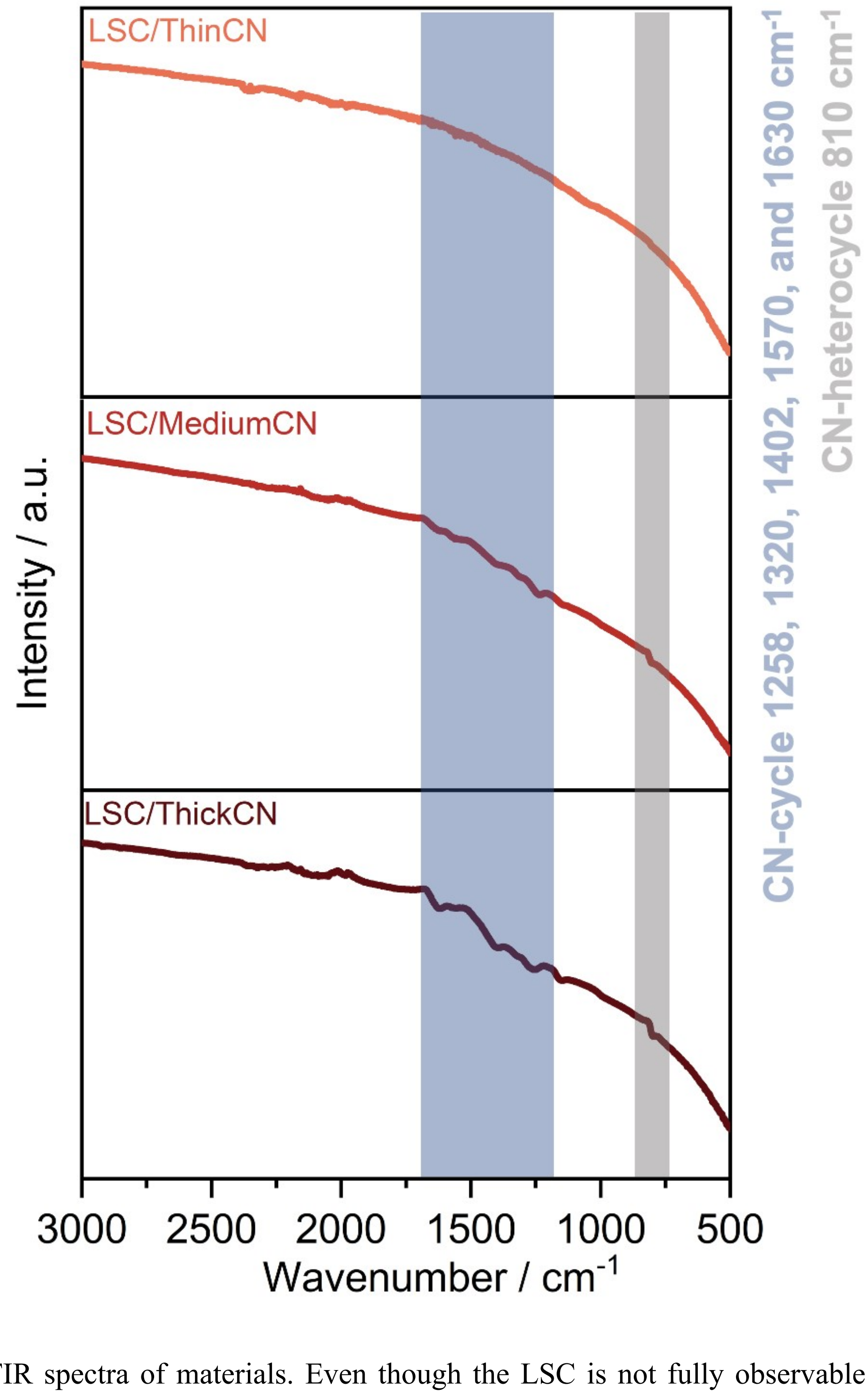

Figure S13. FTIR spectra of materials. Even though the LSC is not fully observable at the IR range, increasing the thickness introduces the typical CN peaks (CN-cycle and CN-heterocycle (heptazine breathing mode)).[3, 12, 13]

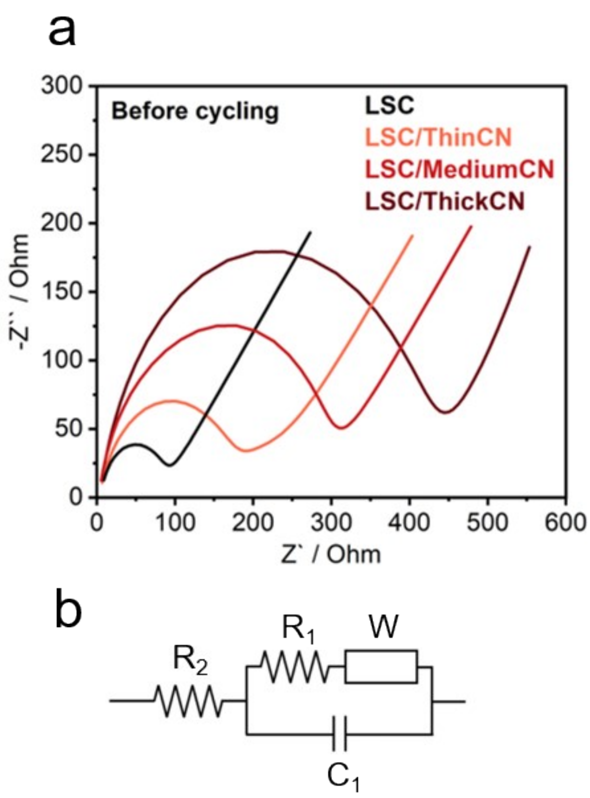

Figure S14. (a) Nyquist plots of the materials before cycling to see the effect of the CN film on charge transfer resistance. (b) Randles circuit model was used to evaluate internal resistance where R_2 is the resistance associated with the electrolyte and electrode, whereas R_1 is related to the charge transfer of the electrode (mainly from LSC/CN film as there is no formation of natural solid electrolyte interphase (SEI) layer at this stage).

Table S2. Internal and charge transfer resistivity of the materials from fitted Randles circuit model.

**Sample**	**R_2 (Ohm cm^{-2})**	**R_1 (Ohm cm^{-2})**
LSC	6.9	86.8
LSC/ThinCN	6.0	158.1
LSC/MediumCN	5.1	266.4
LSC/ThickCN	5.8	396.5

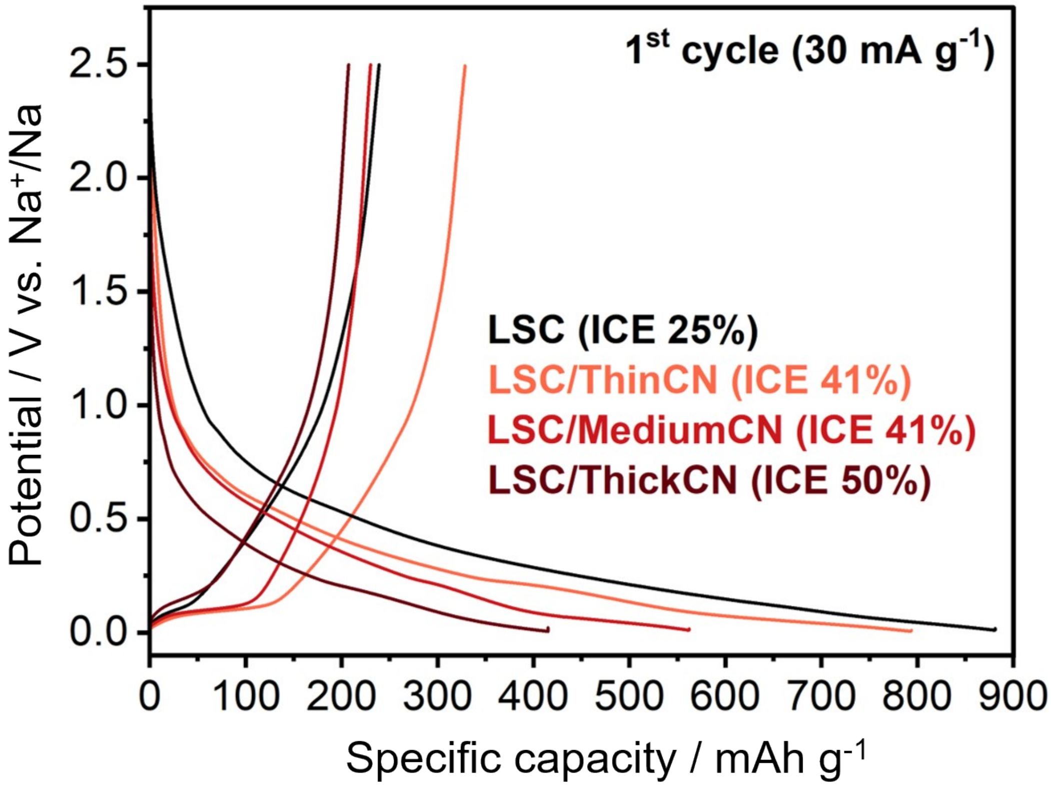

Figure S15. The first cycles of the half-cells with initial Coulombic efficiencies (ICEs).

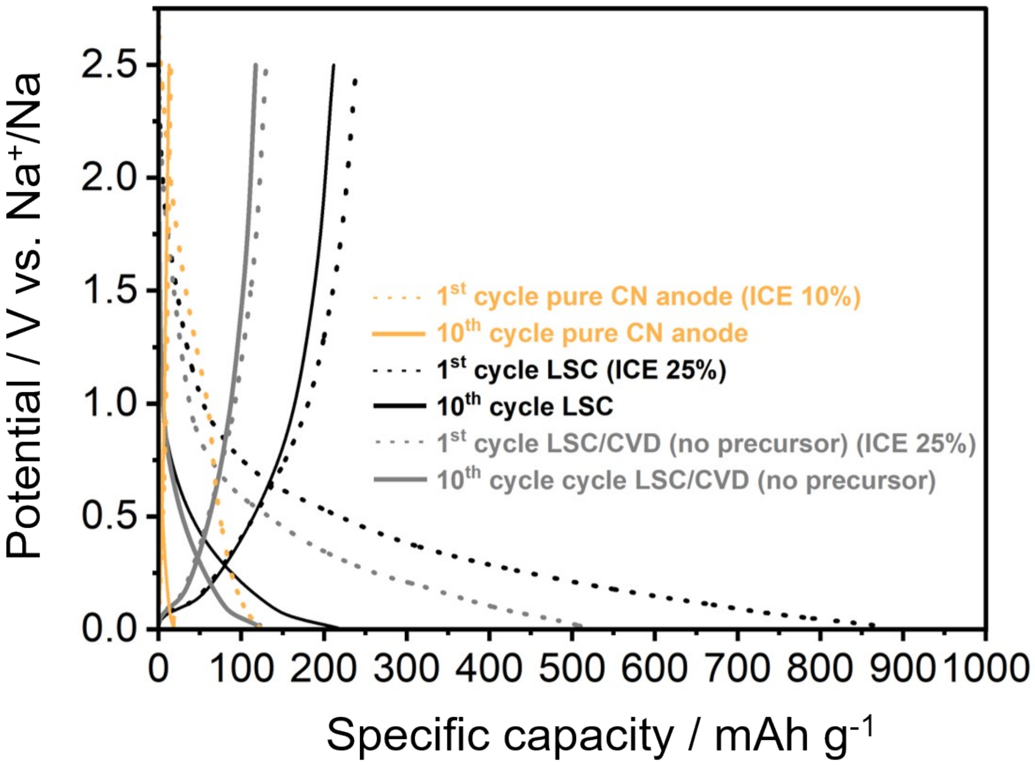

Figure S16. 1st and 10th GCD curves of the pure CN, LSC, and LSC/CVD (without melamine precursor).

Supporting note 5. To prove that the improvement in sodium storage performance is a matter of conformal CN coating, we exfoliated the pristine LSC with the post-treatment in CVD without melamine precursor to see the heat treatment effect on electrochemical performance. By doing so, we observe that heat treatment even decreases the capacity of the pristine LSC, keeping the ICE same, confirming once more the significant improvement in electrochemical performance is due to the conformal CN layer. Moreover, pure bulk CN shows a negligible contribution to sodium energy storage (Figure S16) as well as high electrical resistivity, thus making it an incompatible active material for sodium storage under the same electrochemical conditions. This points further to a synergetic contribution between the LSC/CN rather than the properties of the single material.

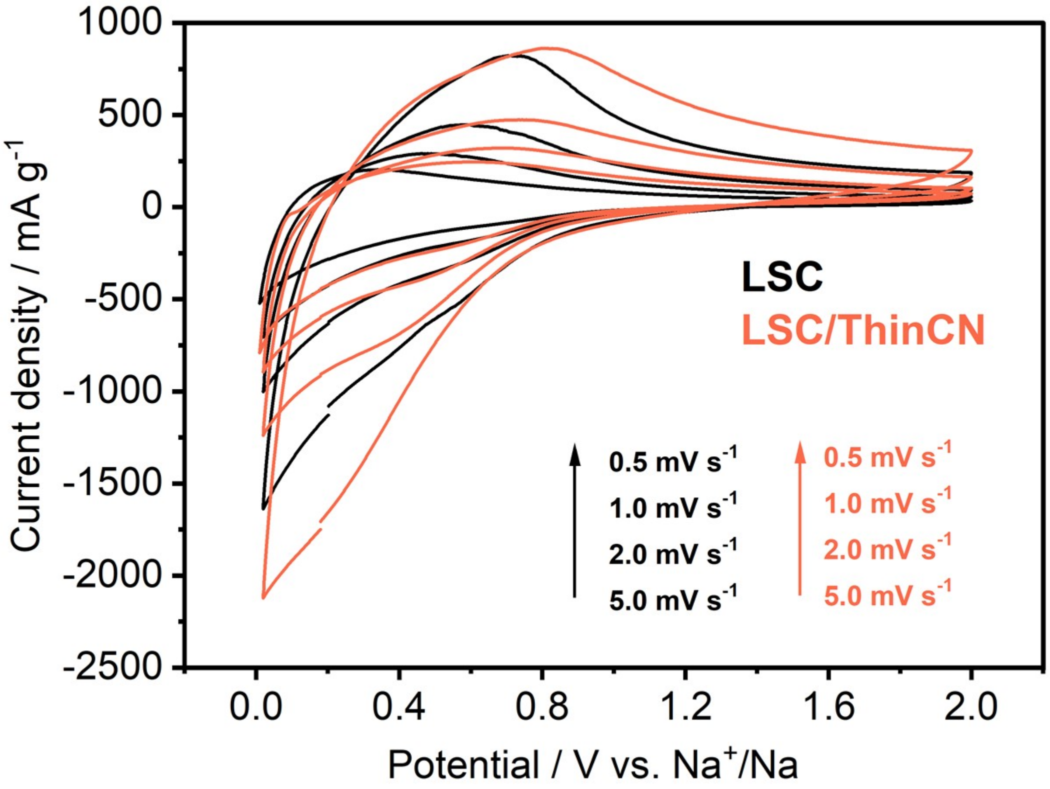

Figure S17. CV plots of LSC and LSC/ThinCN with different scan rates.

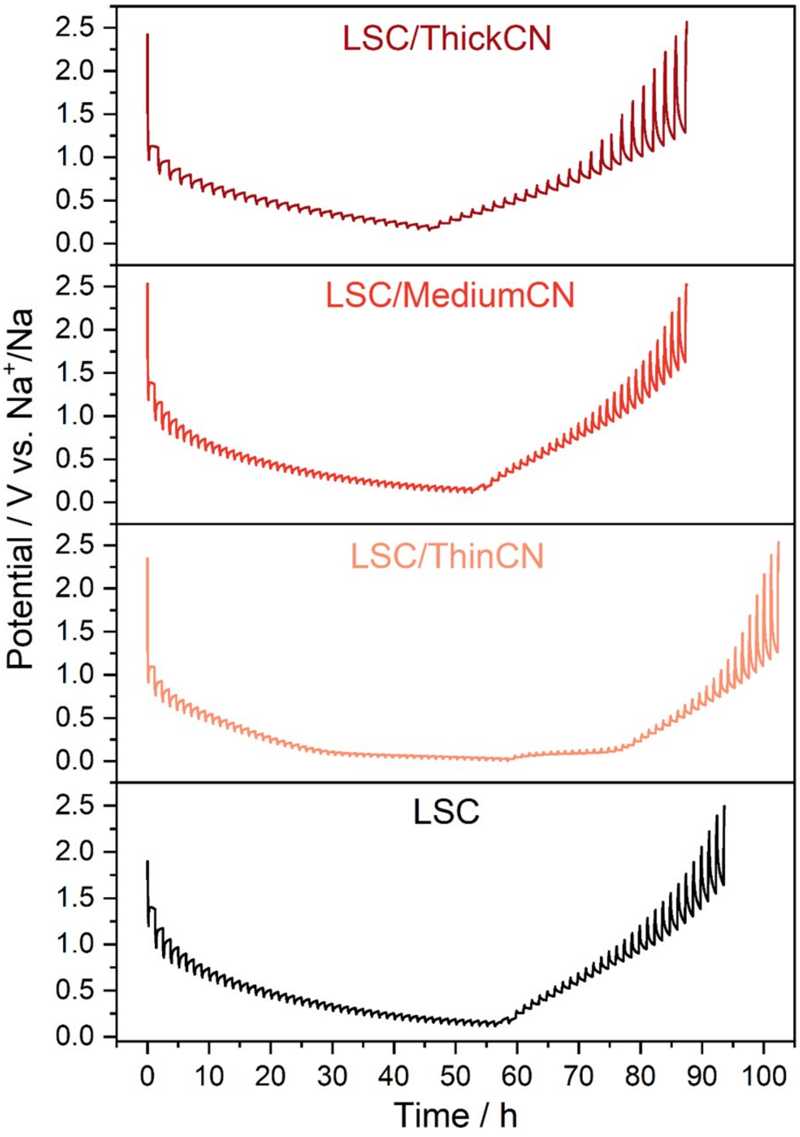

Figure S18. GITT profiles of the electrodes during sodiation and desodiation at 30 mA g^{-1}.

Supporting note 6. We confirmed that the potential response behaves linearly with respect to the square root of the step time (Figure S19). Hence, we used the first-order approximation derived by Weppner and Huggins,[14] which is valid for both spherical and planar geometries.[15] Still, this approach requires a series of assumptions;[16]

i. Step time is significantly less than the effective diffusion time.

ii. Transient data must be large enough not to include ohmic and kinetic overpotential.

Based on the assumptions, diffusion coefficients from GITT measurements can be calculated using the following simplified equation (eq. 1).

$$D_{Na} = \frac{4}{\pi\tau}\left(\frac{m_B V_M}{M_B S}\right)^2 \left(\frac{\Delta E_s}{\Delta E_\tau}\right)^2 \tag{1}$$

Where τ is the pulse duration, m_B and M_B are the actual and molar mass of the active material, V_M is the molar volume, and S is the surface area of the electrodes. Hence, $\left(\frac{m_B V_M}{M_B}\right)$ represents the electrode volume, and dividing it by the S gives electrode thickness. ΔE_s (change of the steady-state voltage during a single-step GITT experiment) and ΔE_τ (change of cell voltage during a constant current pulse) can be extracted from the typical GITT curve of the material (Figure S18).[17, 18] The diffusion coefficients were calculated from 1.0 V to 0.2 V (vs. Na^+/Na) with a step size of 0.05 V (Figure 3e).

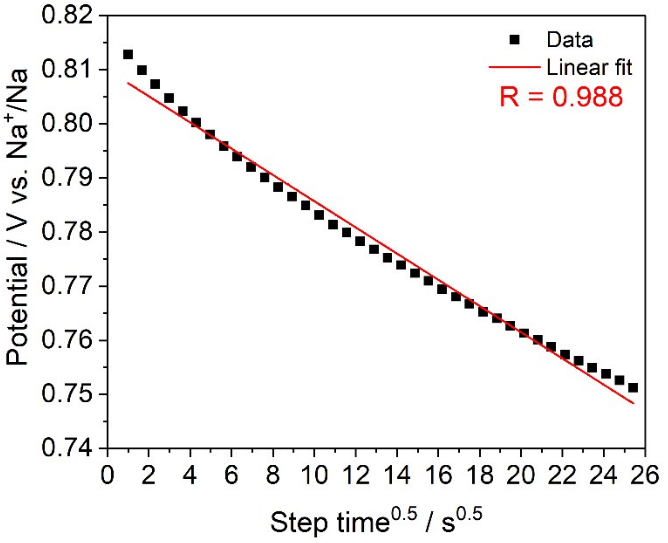

Figure S19. V vs. $s^{0.5}$ plot showing linear fit to simplify Weppner and Huggins model.

Supplementary references

1. F. Brandi, M. Bäumel, V. Molinari, I. Shekova, I. Lauermann, T. Heil, M. Antonietti and M. Al-Naji, *Green Chem.*, 2020, **22**, 2755-2766.
2. M. Al-Naji, F. Brandi, B. Kumru and M. Antonietti, *ChemCatChem*, 2022, **n/a**.
3. P. Giusto, D. Cruz, T. Heil, H. Arazoe, P. Lova, T. Aida, D. Comoretto, M. Patrini and M. Antonietti, *Adv. Mater.*, 2020, **32**, e1908140.
4. N. Soltani, E. Saion, M. Z. Hussein, M. Erfani, A. Abedini, G. Bahmanrokh, M. Navasery and P. Vaziri, *Int. J. Mol. Sci.*, 2012, **13**, 12242-12258.
5. B. D. Fahlman, in *Materials Chemistry*, Springer Netherlands, Dordrecht, 2007, DOI: 10.1007/978-1-4020-6120-2_7, ch. Chapter 7, pp. 357-432.
6. F. Fina, S. K. Callear, G. M. Carins and J. T. S. Irvine, *Chem. Mater.*, 2015, **27**, 2612-2618.
7. P. Giusto, B. Kumru, J. Zhang, R. Rothe and M. Antonietti, *Chem. Mater.*, 2020, **32**, 7284-7291.
8. J. Liu, H. Wang and M. Antonietti, *Chem. Soc. Rev.*, 2016, **45**, 2308-2326.
9. W. Li, M. Zhou, H. Li, K. Wang, S. Cheng and K. Jiang, *Energy Environ. Sci.*, 2015, **8**, 2916-2921.
10. L. Qie, W. Chen, X. Xiong, C. Hu, F. Zou, P. Hu and Y. Huang, *Adv. Sci.*, 2015, **2**, 1500195.
11. T. Wu, M. Jing, L. Yang, G. Zou, H. Hou, Y. Zhang, Y. Zhang, X. Cao and X. Ji, *Adv. Energy Mater.*, 2019, **9**, 1803478-1803478.
12. M. Shen, L. X. Zhang, M. Wang, J. J. Tian, X. X. Jin, L. M. Guo, L. Z. Wang and J. L. Shi, *J. Mater. Chem. A*, 2019, **7**, 1556-1563.
13. M. Kim, S. Hwang and J. S. Yu, *J. Mater. Chem.*, 2007, **17**, 1656-1659.
14. W. Weppner and R. A. Huggins, *J. Electrochem. Soc.*, 1977, **124**, 1569.
15. C. Delacourt, M. Ati and J. M. Tarascon, *J. Electrochem. Soc.*, 2011, **158**, A741.
16. J. S. Horner, G. Whang, D. S. Ashby, I. V. Kolesnichenko, T. N. Lambert, B. S. Dunn, A. A. Talin and S. A. Roberts, *ACS Appl. Energy Mater.*, 2021, **4**, 11460-11469.
17. E. Deiss, *Electrochim. Acta*, 2005, **50**, 2927-2932.
18. Y. Li, Y.-S. Hu, M.-M. Titirici, L. Chen and X. Huang, *Adv. Energy Mater.*, 2016, **6**, 1600659.
